# An investigation into the use of *Caenorhabditis elegans* as a model organism for nerve agent exposure research

**DOI:** 10.1016/j.neuro.2026.103453

**Published:** 2026-05

**Authors:** Johanna Haszczyn, A. Christopher Green, Lindy Holden-Dye, Vincent O'Connor, James Kearn

**Affiliations:** a School of Biological Sciences, Faculty of Environmental and Life Sciences, University of Southampton, Southampton, United Kingdom; b Dstl, Defence Science and Technology Laboratory, Porton Down, Salisbury, Wiltshire, United Kingdom

**Keywords:** *C. elegans*, Nerve agent, Acetylcholinesterase, Soman, Sarin, VX, Organophosphate

## Abstract

Nerve agents exert their toxicological effects via inhibition of acetylcholinesterase (AChE). This inhibition results in excessive cholinergic signalling, which can result in fatality through cessation of breathing. Current therapies for nerve agent poisoning antagonise the overstimulation of muscarinic acetylcholine receptors and use benzodiazepines to enhance GABAergic inhibition as a counter to increased excitability in the central nervous system. *Caenorhabditis elegans* (*C. elegans*) can be effectively used to assess the toxic effects of AChE inhibition by paraoxon-ethyl, which shares the same mode of action of AChE inhibition as organophosphate (OP) nerve agents. In particular, the *C. elegans* behaviours pharyngeal pumping and motility can serve as bioassays of neuromuscular function and intoxication in the intact animal. This is reinforced by measuring the OP inhibition of AChE activity from homogenates of treated worms. Here, we demonstrate that paraoxon-ethyl, sarin, soman and VX elicit an irreversible inhibition of AChE activity with an order of potency sarin>soman>paraoxon-ethyl>VX. Importantly, this order of potency is maintained when pharyngeal pumping and motility are used to assess nerve agent exposure *in vivo*. *C. elegans* neuromuscular-dependent behaviours recover following transfer to OP-free plates, which is not observed for biochemical *in vitro* AChE activity. Interestingly, we see recovery of extracted *in vitro* AChE activity following removal of the intoxicated worms to OP-free plates. These data support the use of *C. elegans* to model nerve agent inhibition and recovery. In particular, the molecular mechanism(s) underpinning behavioural recovery merit further investigation.

## Highlights

• *C. elegans* acetylcholinesterase (AChE) is irreversibly inhibited by nerve agents.
• The potency of sarin and soman is similar to that seen for mammalian AChE activity.
• Inhibition of AChE correlates with inhibition of *C. elegans* pharyngeal pumping.
• Pharyngeal pumping recovers following removal of the organophosphate.
• Body wall muscle nAChR mutants are less sensitive to inhibition of pharyngeal pumping.

## 1 Introduction

**Fig. 1:**
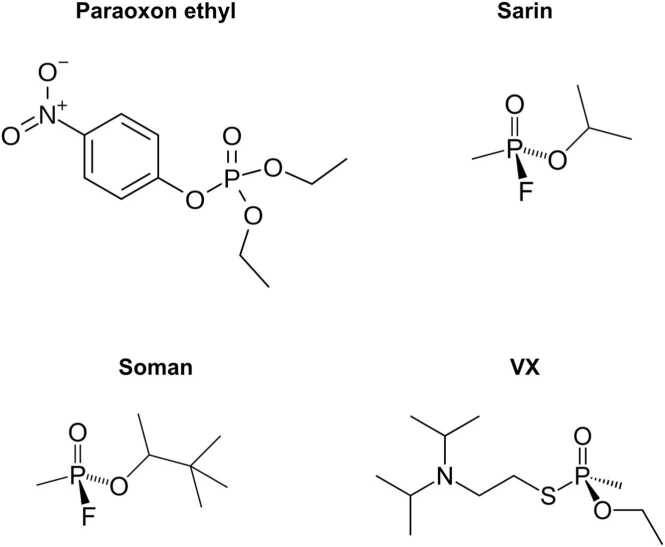
The structures of the organophosphate paraoxon ethyl and the nerve agents sarin, soman and VX.

Nerve agents are potent inhibitors of AChE and exposure results in elevation of acetylcholine beyond normal physiological levels (Fig. 1). This results in sustained over-stimulation of nicotinic and muscarinic acetylcholine receptors in the peripheral and central nervous systems (Wille et al., 2019). These organophosphate (OP) nerve agents, including sarin, soman and VX, have been used as chemical weapons in several conflicts and in assassination attempts (Salem et al., 2019). Current treatments include oximes, which reverse the initial covalent modification between the OP and the AChE by hydrolysis (Amend et al., 2020, Hulse et al., 2019). This reaction must compete against the secondary reaction known as aging, in which the initial OP-AChE covalent modification is further dealkylated to generate a negatively charged complex, which becomes insensitive to nucleophilic oxime reactivation. These competing inhibition and mitigation reactions are very dependent on the structure-function relationships of the individual OP with AChE (Worek et al., 2004). For instance, when the aging situation is predominant, recovery is dependent on the metabolic turnover of the inhibited enzyme. This supports the idea that improvements to current therapies or alternative ways to support neuromuscular transmission at poisoned synapses that are independent of AChE re-activation are imperative (Worek et al., 2020).

Previously, it has been shown that the feeding behaviour pharyngeal pumping and *C. elegans* movement are readily tractable bioassays of cholinergic function that can be used to investigate OP intoxication using the pesticide paraoxon-ethyl (Izquierdo et al., 2021). In particular, pharyngeal pumping was shown to be an effective read out of body wall muscle function, with the explanation being that hypercontraction of the worm causes a distal inhibition of feeding (Izquierdo et al., 2021). The more striking observation in this analysis was that a worm fully exposed to OP and subsequently removed could gain full recovery in its cholinergic-dependent function. This highlights the potential to investigate *in vivo* recovery in absence of the confound of organism mortality (Izquierdo et al., 2023). In mammals, OP poisoning can be fatal as a result of bronchial constriction, disruption of central respiratory drive and paralysis of respiratory muscles. The lack of fatality in *C. elegans* is largely because it does not require neuromuscular activity to support respiration. In addition, the use of *C. elegans* for recovery studies was reinforced by showing that oxime treatment can accelerate the recovery of OP sensitive behaviours (Izquierdo et al., 2023). Importantly, these *in vivo* studies were supported through measurement of OP inhibition of AChE enzyme activity found in homogenates of *C. elegans*. Overall, this work demonstrated that *C. elegans* are a suitable model for OP studies.

This is the first investigation to evaluate the advantages and limitations of using *C. elegans* as a model for nerve agent exposure research. Specifically, we compared the potency of the nerve agents sarin, soman and VX, benchmarked against paraoxon-ethyl on *in vivo* measures, using *C. elegans* pharyngeal pumping as a readout. In parallel, we determined the rank order of potency of these agents *in vitro* against *C. elegans* AChE benchmarked against rat AChE. This work aims to support future investigations that can utilise *C. elegans* as a genetically tractable paradigm to explore routes to mitigate human OP poisoning.

## 2 Materials and methods

### 2.1 *C. elegans* maintenance

The N2 wild-type, CB193 *unc-29* (*e193*), CB211 *lev-1* (*e211*) *C. elegans* strains (Table 1, Table 2) were obtained from the *Caenorhabditis* Genetics Centre and were maintained under standard conditions (Brenner, 1974). *C. elegans* were grown at 20 °C on Nematode Growth Medium (NGM) agar plates seeded with *E. coli* OP50 as a source of food.

**Table 1:** Mutant strains used, including their mutation position and predicted consequence. TM = Trans-membrane domain.

**Strain**	**Gene**	**Allele**	**Given name**	**Region**	**Predicted consequence**	**Reference**
CB193	*unc-29*	*e193*	UNC-29-TM2-TM3-P272S	TM2-TM3 loop	*C. elegans* mutant receptors in Xenopus oocytes reduced current amplitude of the receptor. The mutation P272S impairs gating by reducing the gating equilibrium by 150-fold.	(Izquierdo et al., 2023, Lee and Sine, 2005)
CB211	*lev-1*	*e211*	LEV-1-TM4-G461E	TM4	*C. elegans* mutant receptors in Xenopus oocytes reduced current amplitude of the receptor. Other work suggested reduced membrane interactions and can alter desensitization kinetics.	(Domville and Baenziger, 2018, Izquierdo et al., 2023)

**Table 2:** **Comparison of inhibition of*****C. elegans*****AChE activity relative to rat brain using paraoxon-ethyl and nerve agents.** The IC_50_ and number of replicates for each experiment shown in Fig. 1 are presented. Results are from 3 independent experiments each conducted in duplicate.

	**Rat**		***C. elegans***	
OP	**IC**_**50**_**(nM)**	**IC**_**50**_**95% CI (nM)**	**IC**_**50**_**(nM)**	**IC**_**50**_**95% CI (nM)**
Paraoxon-ethyl	46.7	40–54	95.4	49–119
Sarin	1.8	1.7–2.1	5.7	37–63
Soman	3.8	3.2–4.4	9.2	7.5–11
VX	2.3	2.6–3.2	131.6	99–180

### 2.2 Chemical stocks

Paraoxon-ethyl (1 M) (Merck) was supplied dissolved in 100% DMSO. Nerve agents (10 mM sarin, 10 mM soman and 10 mM VX) were synthesised and provided by DSTL, Porton Down (UK) and dissolved in 100% iso-propyl-alcohol (IPA). The OP inhibitor stocks were kept at −80 °C. Acetylthiocholine (ATCh) and 5,5′-dithio-bis-2-nitrobenzoic acid (DTNB) were obtained from Merck.

### 2.3 OP NGM plate preparation

Experiments were performed in 6-well plates containing a final NGM volume of 3 ml. OP containing plates were prepared the day before by adding an aliquot of a concentrated OP inhibitor stock into the melted NGM to give the indicated final concentration. *E. coli* OP50 bacteria (50 μl) was added on the plate when the media was solidified. After 1 h in a fume hood, the dried bacterial plates were sealed and kept in the dark at 4 °C until next day.

Plates were left at room temperature for at least 30 min before starting the experiment. The final concentration of vehicle in the behavioural assay was 0.1% of DMSO for paraoxon-ethyl and 0.5% of IPA for OP nerve agent plates. Control plates contained the same concentration of vehicle as assay plates. Neither vehicle at these concentrations had any effect on the phenotypes tested.

### 2.4 Behavioural assays

*C. elegans* were viewed under a Nikon SMZ800 binocular zoom microscope. L4 (4th larval stage) worms were recognised by the vulva saddle and were age-synchronised through selection 16 −24 h before the experiment by moving to fresh OP50-seeded plates. All behavioural experiments described here were performed on synchronised L4 + 1-day old adults (L4 + 1) at room temperature (20 °C).

### 2.5 Pharyngeal pumping

Pharyngeal pumping on food was scored at indicated times after transferring worms to OP or vehicle control plates. *C. elegans* that left the patch of food during the experiment were picked back to the bacterial lawn and their pump rate recorded 10 min after return to food. Pumping was quantified by counting the number of grinder movements observed under a binocular microscope using a handheld counter. The number of pumps was recorded for a minimum of 2 min per worm at each time point and the pump rate is plotted as pumps per minute.

### 2.6 Body-Length

Body length was measured as previously described (Izquierdo et al., 2021, Mulcahy et al., 2013). Briefly, images of the worms were acquired using a Nikon SMZ800 binocular zoom microscope. These images were binarized and skeletonized using ImageJ software. The length of the skeleton was used to determine the body length of the nematodes. Percentage reduction in body length was calculated relative to the body length of each worm pre-exposure.

### 2.7 Organophosphate intoxication and recovery

To investigate the recovery of pharyngeal pumping following OP intoxication, L4 + 1 worms were put on OP inhibitor-treated plates at room temperature for 24-hours. The lowest concentration that gave the maximal inhibition of pumping after a 24-hour exposure was used (Izquierdo et al., 2023). After 24-hours, the exposed *C. elegans* were transferred on to an OP inhibitor-free plate containing a lawn of *E. coli* OP50. The recovery of pharyngeal pumping from full inhibition was measured by recording the pharyngeal pump rate up at indicated intervals over 24-hours.

### 2.8 Preparation of rat brain and *C. elegans* homogenates

A synchronised population of eggs was generated from 20 5.5 cm plates with a mixed population of worms by bleaching (Hibshman et al., 2021). Bleached eggs were placed on 9 cm plates with no food for 24-hours to generate a synchronised populations of L1 arrested worms. L1 worms were transferred on to 9 cm plates containing a saturated lawn of *E. coli* (300 μl) and allowed to develop to L4. L4 worms were checked for the presence of their vulval saddle. These worms were allowed to develop to L4 + 1 (20 °C). This generated around 10,000 worms which were harvested and washed three further times with 0.1 M phosphate buffer pH 8.0 to remove all the remaining bacteria. *C. elegans* were transferred to a glass homogenizer and incubated for 30 min on ice with a final concentration of 0.15% of n-octyl-glucoside (w/v) as detergent to permeabilise the cuticle and release cellular contents (Blaxter, 1993). *C. elegans* were then homogenised in the glass homogeniser by hand for 20 min on ice. The disruption was monitored by visual inspection under a microscope for ghost-like worms.

Rat brain tissue was provided by Dstl as spare tissue from unrelated experiments and was stored at −80 °C until time of use. Rat brain tissue, in phosphate buffer (w/v) with 0.15% of n-octyl-glucoside, was homogenised using glass homogenisers in the same way as *C. elegans*. The resulting worm and rat homogenates were stored at −80 ᵒC until use.

### 2.9 AChE activity in *C. elegans* and rat brain homogenates

For use in AChE assays, homogenates were defrosted on ice. A standard Bradford assay was then used to measure their protein content (QuickStart™ Bradford Protein Assay, Bio Rad).

AChE activity was measured using a modified colorimetric Ellman’s assay (Ellman et al., 1961, Izquierdo et al., 2021). The assay mixture contained 100 µg of homogenate protein in a final volume of 200 µl of 0.1 M phosphate buffer pH 8.0 which including 0.48 mM acetylthiocholine (ATCh) as substrate and 0.32 mM 5,5-dithio-bis-(2-nitrobenzoic acid) (DTNB). The increase in absorbance at 412 nm was measured at 1 min intervals for 15 min at room temperature using a FlexStation® 3 plate reader. The change in absorbance against time due to the production of 5-nitro-2-thiobenzoic acid (TNB^2-^) was used to calculate the AChE activity. OP inhibitor-treated AChE activity was calculated as a percentage of the rate of change in the absence of OP inhibitor. The data were corrected using the background absorbance of 1) ATCh in the absence of protein and 2) protein signal in the absence of ATCh which were run as controls.

For experiments with AChE inhibitors, a concentration-range was prepared in a 96-well plate. 50 μl aliquots of AChE inhibitor were added to 50 μl of homogenate (total 100 μg homogenate protein) and 100 μl of the reaction mixture (ATCh, DTNB and 0.1 M phosphate buffer pH 8.0) to give a final volume of 200 μl. Absorbance was measured for 15 min at room temperature. The rate of change in absorbance was measured and AChE activity in the presence of AChE inhibitor was calculated as a percentage of the rate of change in the absence of inhibitor.

### 2.10 Measuring AChE recovery of activity following exposure to AChE inhibitors

OP-inhibited AChE was prepared by incubating 2.4 mg/ml of *C. elegans* homogenate with the AChE inhibitor. The concentration of inhibitor was calculated based on estimating the lowest concentration that gave the maximal inhibition of AChE activity after 15 min (paraoxon-ethyl = 1 μM, sarin = 50 nM, soman = 100 nM, VX = 1 μM). Rat and *C. elegans* homogenates were incubated with the specified concentration of OP inhibitor or vehicle for 30 min at room temperature to allow full inhibition. The mixture was centrifuged at 14,000 rpm for 10 min and the supernatant was discarded to remove the excess of OP. The pellet containing the untreated or washed OP inhibitor-treated homogenate was resuspended to an equal volume with 0.1 M phosphate buffer pH 8.0 and incubated at room temperature (20 °C). The predicted concentration of residual inhibitor following washing was (paraoxon-ethyl ≈ 100 nM, sarin/soman/VX ≈ 0.4–1 nM) in the assay. These concentrations would allow for 90% activity. AChE activity in the control and washed homogenates was measured at indicated times (0-, 1-, 3- and 6-hours). AChE activity was calculated as a percentage of the rate of change in the absence of OP inhibitor.

### 2.11 AChE activity measured from whole worm homogenates after exposure and recovery from paraoxon-ethyl

AChE activity was measured in worm homogenates that were taken from worms across an exposure time course, and after recovery. A collection of 500 L4 + 1 *C. elegans* were transferred on to duplicate plates containing vehicle or AChE inhibitor and left for 24-hours. Treated plates contained AChE inhibitor at a concentration that caused a maximal inhibition of pharyngeal pumping (500 μM). After a 24-hour incubation at room temperature, the control and OP inhibitor-exposed worms were harvested, washed three times with 0.1 M sodium phosphate buffer pH 8 (by resuspension and centrifugation) and homogenised.

The groups of worms that were used to measure AChE recovery following OP exposure were transferred to OP inhibitor-free plates by washing them off with 5 ml M9 (3 g KH2PO4, 6 g Na2HPO4, 5 g NaCl, 1 ml 1 M MgSO4, H2O to 1 litre. Produced in house and sterilised by autoclaving), followed by three further washes in 10 ml of 0.1 M sodium phosphate buffer pH 8. The supernatant was discarded, and the remaining pellet of worms (∼100 μl) was transferred to the food lawn of control plates. This population of *C. elegans* were allowed to recover for 24-hours. After 24-hours, *C. elegans* were harvested and homogenised.

The homogenates from before, during and following AChE inhibitor exposure, were used in an Ellman assay to measure AChE activity. AChE activity was calculated as a percentage of the rate of change in the absence of OP inhibitor.

### 2.12 Data and statistical analysis

The data were analysed using GraphPad Prism (GraphPad Prism for Windows version 10.4.2 www.graphpad.com) and are displayed as the mean ± SEM. Statistical significance was measured using either a one-way or two-way ANOVA followed by Bonferroni post-hoc analysis. Bonferroni corrections were selected to avoid false positives. The sample size N of each experiment is specified in figures legends.

AChE activity in this work was represented as the increase in absorbance over time. The concentration-response analysis for inhibition potency comparisons were calculated using GraphPad Prism 9. The change in absorbance of homogenates exposed to increasing concentrations was plotted as a percentage of control. The concentrations were transformed (log_10_), and a non-linear regression (curve fit) of a log(inhibitor) vs. response – variable slope (four parameters) was used to fit the concentration-response curve. The curves were fitted (top=100, bottom=0). This calculated the IC_50_, slope and the 95% CI of the fitted parameters.

## 3 Results

### 3.1 A comparison of organophosphate nerve agent inhibition of *C. elegans* and mammalian AChE

This is the first documented work that has used *C. elegans* as a model for nerve agent exposure. To assess whether *C. elegans* AChE is inhibited by nerve agents, homogenate of *C. elegans* and mammalian rat brain was treated with OPs to measure and compare inhibition of AChE activity between species. The concentration-dependent inhibition of *C. elegans* AChE activity was measured following paraoxon-ethyl exposure, and this was compared to the nerve agents sarin, soman and VX to allow comparison of relative potency. A modified Ellman’s assay was used, where the homogenates were exposed for 20 min and activity was measured in the absence and presence of AChE inhibitor (Ellman et al., 1961, Izquierdo et al., 2021).

Rat brain AChE activity was reduced in the presence of increasing concentrations of paraoxon-ethyl, sarin, soman and VX. AChE activity was reduced to around 0% of the control value at 1 μM for paraoxon-ethyl and 100–200 nM in the nerve agents sarin, soman and VX (Fig. 1). The clear increase in potency to AChE inhibition for the nerve agents relative to paraoxon-ethyl is consistent with previous reports of potency seen with mammalian AChE, with sarin ≈ VX > soman > paraoxon-ethyl (Table 1) (Franjesevic et al., 2019, John et al., 2020, Worek et al., 2004).

When *C. elegans* homogenates were exposed to paraoxon-ethyl or nerve agents*,* AChE activity was also reduced in a concentration-dependent manner. Sarin and soman were more potent relative to paraoxon-ethyl, which was similar to the rat brain AChE activity results. In contrast, VX had a relatively reduced potency in *C. elegans*, with a potency ∼50-fold lower than that seen for rat brain homogenate. Additionally, VX exhibited a marked reduction in its ability to inhibit AChE compared to sarin and soman. This shifted the rank order of potency of the OP and OP nerve agent series studied here such that sarin ≈ soman > paraoxon-ethyl > VX, in contrast to rat brain. This indicates that under these experimental conditions, *C. elegans* AChE is less sensitive to VX compared to that expressed in rat brain.

### 3.2 *C. elegans* AChE is irreversibly inhibited by organophosphates *in vitro*

**Fig. 2:**
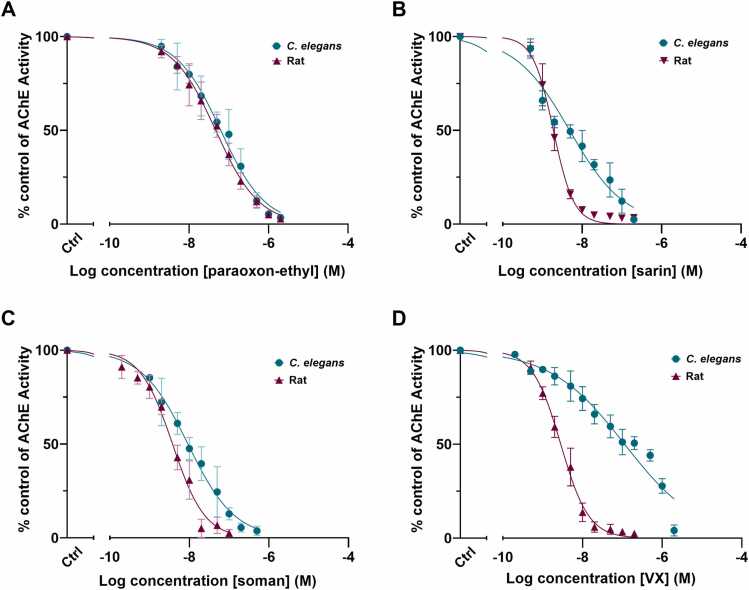
**OP nerve agents inhibit*****C. elegans*****and rat AChE activity with a similar rank order of potency, with the exception of VX.***C. elegans* homogenate or rat brain homogenate AChE activity was quantified in the presence or absence of a concentration range of paraoxon-ethyl, sarin, soman or VX. Untreated homogenates were used as controls. AChE activities are represented as the percentage of activity relative to the untreated control. Rat and *C. elegans* homogenates were inhibited by paraoxon-ethyl (**A**), sarin (**B**), soman (**C**) and VX (**D**). The data represented three experiments in duplicate for each inhibitor for both worm and rat (*n* = 3). Data was plotted as a % of control values (log(inhibitor) vs. response four parameter variable slope) and fitted (top=100, bottom = 0).

To establish whether the inhibitory effect of OPs on *C. elegans* AChE was irreversible, *in vitro* washout experiments were performed. For this, *C. elegans* or rat brain homogenate were exposed to a concentration that fully inhibited enzyme activity for each inhibitor for 30 min at room temperature (21 °C). The concentrations used to achieve maximal inhibition were: paraoxon-ethyl = 1 μM, sarin = 50 nM, soman = 100 nM, VX = 1 μM. This inhibited homogenate was then washed before being tested for AChE activity. No recovery of inhibited AChE occurred in homogenates 6-hours after washing out the OP (Fig. 2). This showed that, as with the mammalian enzyme, OPs irreversibly inhibit *C. elegans* AChE *in vitro*.

### 3.3 Pharyngeal pumping as a behavioural output for OP nerve agent AChE inhibition

Having demonstrated that paraoxon-ethyl, sarin, soman and VX are potent inhibitors of *C. elegans* AChE activity, this comparison of AChE inhibitor potency was then extended by utilizing cholinergic-dependent *C. elegans* behaviours to assess nerve agent effects on the live worm. *C. elegans* has a cuticle that could limit access of OP inhibitors into the worm. It was predicted that *C. elegans* nerve agent exposures would require concentrations that exceed those needed for the *in vitro* inhibition described above. For this reason, worms were exposed to a range of concentrations to identify maximal and IC_50_ concentrations, in keeping with what was described for paraoxon-ethyl and aldicarb previously (Izquierdo et al., 2021).

**Fig. 3:**
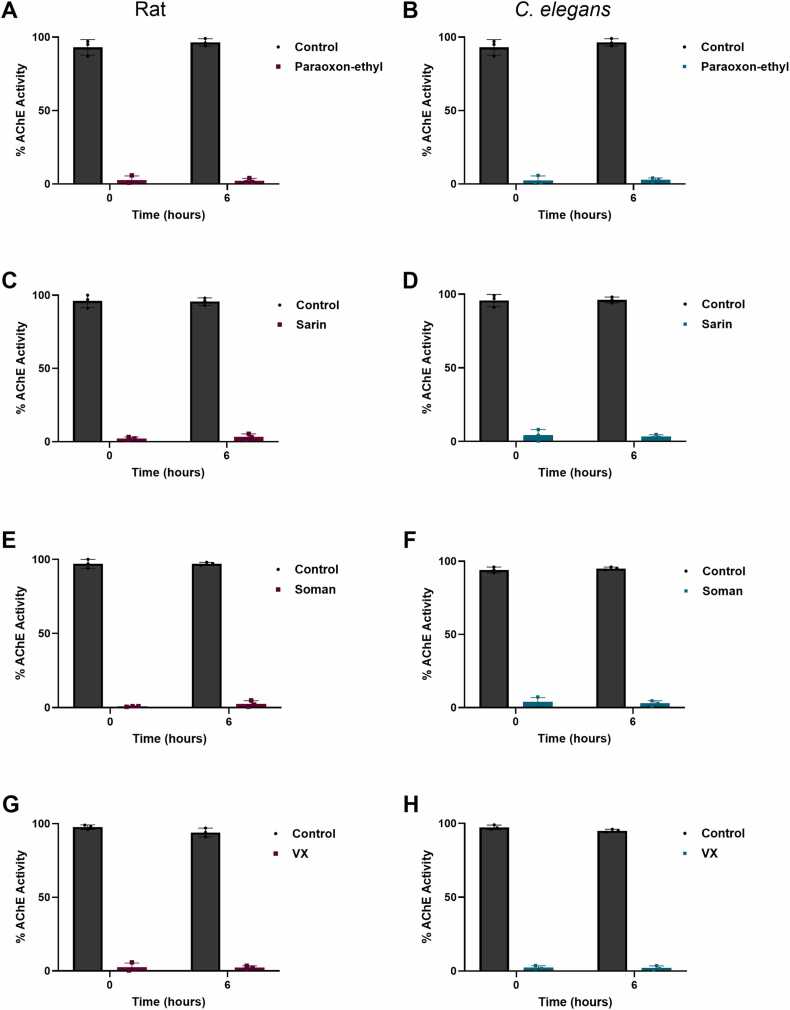
**OP inhibition of homogenate AChE activity does not reverse over 6-hours.** AChE activity was measured after incubating rat brain or *C. elegans* homogenates treated with or without paraoxon-ethyl = 1 μM (**A** and **B**), sarin = 50 nM (**C** and **D**), soman = 100 nM (**E** and **F**), VX = 1 μM (**G** and **H**) for 30 min (0 h). The activity was also measured in homogenates 6-hours after washing the initial control and inhibited homogenates to remove OP. The change in absorbance over time was measured in the presence and absence of OP inhibitor. The AChE activity is represented as the percentage activity after treatment relative to the corresponding untreated control. Data are from three independent experiments in duplicate for each inhibitor for both worm and rat (*n* = 3). There was no statistically significant difference (p > 0.05) between time= 0 and time= 6 in all assays (unpaired student’s *t*-test).

*C. elegans* were exposed to a concentration-range of soman, sarin and VX, which induced a time- and concentration-dependent inhibition of pharyngeal pumping. At the higher concentrations, *C. elegans* exposed to sarin, soman and paraoxon-ethyl exhibited a rapid reduction in pharyngeal pumping that was complete within one hour at 10 μM, 50 μM and 100 μM, respectively. In contrast, VX induced a slower decline at the concentrations tested, where pharyngeal pumping failed to reach complete inhibition at 50 μM, even after 24-hours (Fig. 3). Experiments with higher concentrations of VX could not be conducted due to health and safety restrictions. Estimates of the IC_50_ for inhibition of pharyngeal pumping show that the rank order of potency for these AChE inhibitors was sarin > soman> paraoxon-ethyl > VX, with IC_50_ values of 5.67 μM, 2.55 μM, 43.6 μM and > 50 μM, respectively (Table 3). This rank order of potency was consistent with the *in vitro* enzyme activity assay in Fig. 1, providing evidence that pharyngeal pumping serves as an *in vivo* proxy for OP inhibition of AChE activity in the intact worm.

**Fig. 4:**
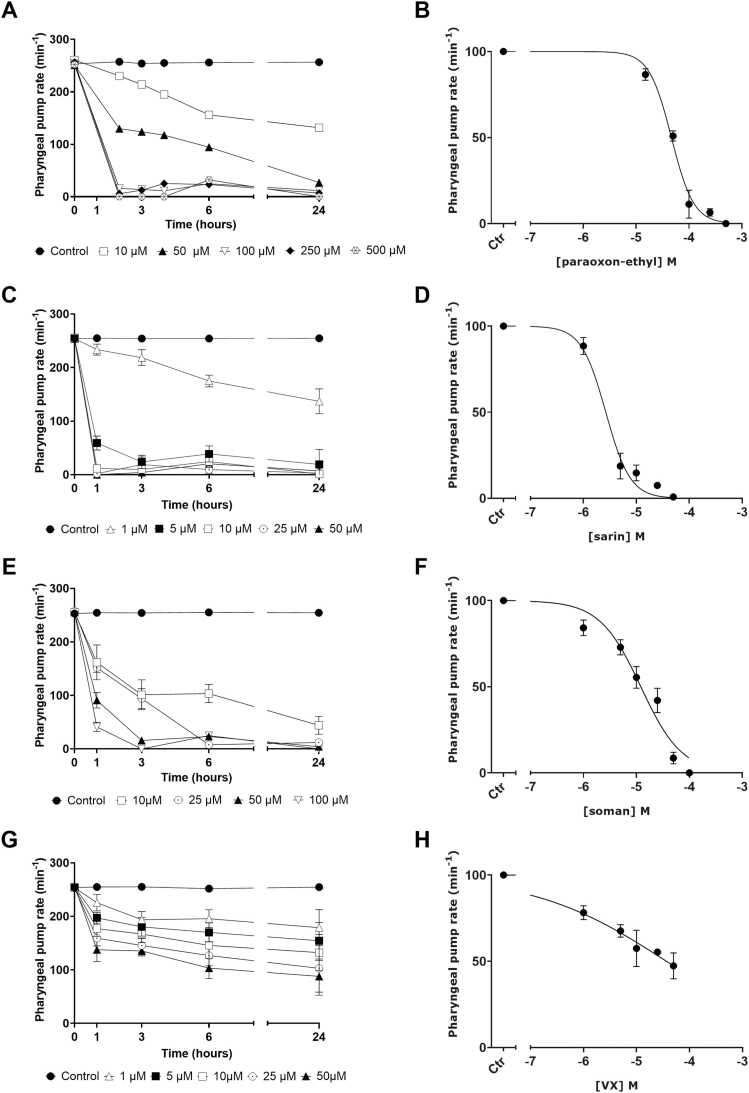
Effect of paraoxon-ethyl and the nerve agents sarin, soman and VX on the pharyngeal pumping rate of N2 worms. Pharyngeal pumping rate at 3-hours was used to generate the plotted concentration-response curves for (**A)** paraoxon-ethyl, (**C**) sarin (**E**) soman and (**G**) VX. For the concentration-response curves, the percentage of the maximum of pharyngeal pumping in each experiment was calculated and plotted as a function of the concentration of each OP inhibitor ((**B**) paraoxon-ethyl, (**D**) sarin (**F**) soman and (**H)** VX). The plot was fitted (top = 100, bottom = 0) and used to estimate IC_50_ and confidence limits (Table 2). N = 3–4 independent experiments, conducted in duplicate.

**Table 3:** Inhibition of *C. elegans* pharyngeal pumping using paraoxon-ethyl and nerve agents. The IC_50_, logIC_50_ and N numbers for each experiment are presented. N = 3–4 independent experiments, conducted in duplicate.

OP	IC_50_ (µM)	IC_50_ 95% CI (µM)
Paraoxon-ethyl	46.6	39.8–53.5
Sarin	2.7	2.1 – 3.3
Soman	12.1	8.9 – 16.1
VX	36.7	22.5 – 82.1

### 3.4 *C. elegans* pharyngeal pumping recovers at differential rates for different OP nerve agents following removal from exposure

**Fig. 5:**
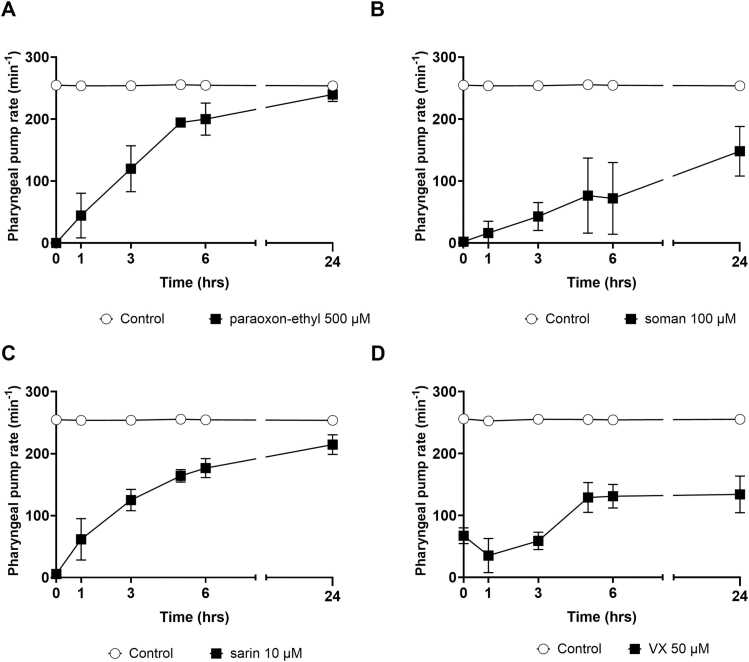
A comparison of pharyngeal pumping recovery rates following OP exposure. A-D) The pharyngeal pumping rate of worms that were allowed to recover on untreated plates after a 24-hour exposure of OP. A maximal concentration that fully inhibits the pharyngeal pump phenotype of each OP inhibitor was used (**B** paraoxon-ethyl = 500 μM, **C** sarin = 10 μM, **D** soman = 100 μM, **E** VX = 100 μM). N = 3–4 independent experiments, conducted in duplicate.

The recovery of *in vivo* behavioural responses was then compared to *in vitro* enzyme activity. As the worms remain viable after prolonged exposure, recovery from inhibition could be used to characterise the effects of OP inhibitors on *C. elegans* and permit comparison with enzyme activity recovery. Pharyngeal pumping was measured in *C. elegans* that were permitted to recover from maximal inhibition by placing them on OP-free plates. This was done by maximally inhibiting pharyngeal pumping at a select concentration (paraoxon-ethyl = 500 μM, sarin = 10 μM, soman = 100 μM, VX = 50 μM) for 24-hours. Worms were then transferred to fresh untreated plates (Fig. 5 A) and their pharyngeal pumping was scored for recovery at distinct time points over 24-hours.

Worms exposed to a maximal concentration and subsequently transferred to control plates exhibited a clear time-dependent recovery in pharyngeal pumping in the worms treated with paraoxon-ethyl, sarin, soman and VX (Fig. 5). Paraoxon-ethyl and sarin showed recovery with a t_1/2_ of approximately two-hours. In contrast, worms exposed to 100 μM soman and 50 μM VX recovered more slowly, with recovery incomplete after 24-hours on OP inhibitor-free plates. In addition, there was no significant difference between recovery at 6- hours and 24-hours, which suggests that maximal total recovery that can be achieved had been achieved by 6-hours. When comparing the *in vitro* total inhibition and lack of recovery (Fig. 5) and the *in vivo* recovery, it highlighted that the *in vivo* situation is more complex.

### 3.5 The recovery of pharyngeal pumping and body length following removal from OP exposure corresponds to increased AChE activity *in vivo*

**Fig. 6:**
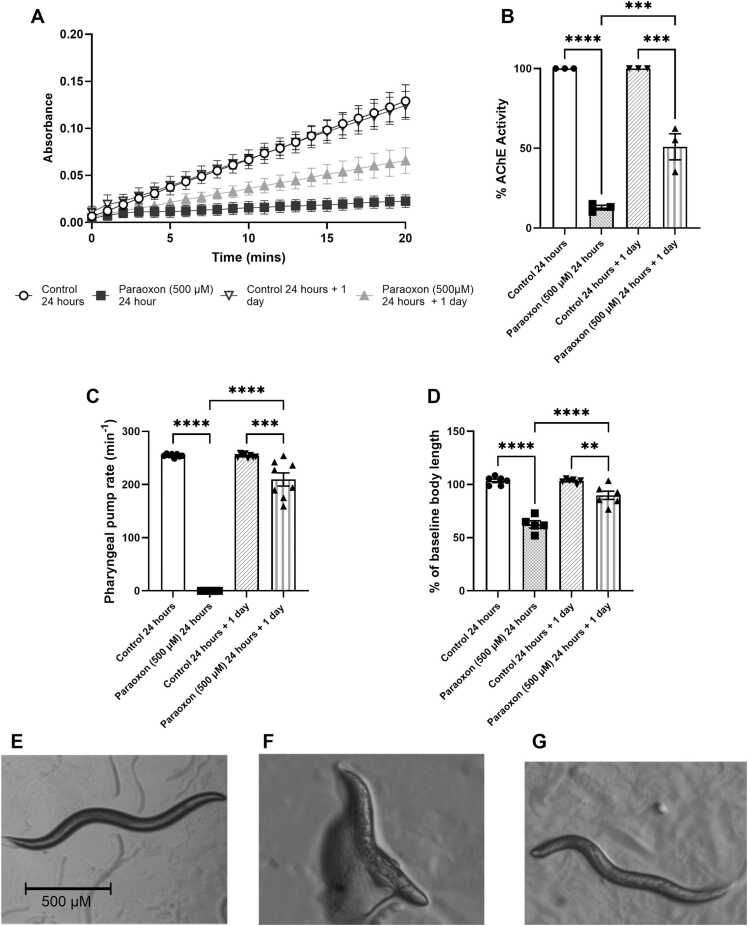
AChE activity and recovery in enzyme extracted from worms after *in vivo* exposure with paraoxon-ethyl. Paraoxon-ethyl inhibited *C. elegans* enzyme activity in the absence and presence of OP inhibitor after 24-hours. Untreated or paraoxon-ethyl-treated *C. elegans* were homogenized after OP inhibitor incubation or following a further 24-hour incubation with recovery. The groups that were allowed to recover were homogenised on the following day. (**A**) Absorbance data in the Ellman assay for different treatments. (**B**) The % recovery of the enzyme activity relative to the untreated control at 24-hours (**C**) Pharyngeal pump rate of *C. elegans* in control, paraoxon-ethyl or following paraoxon-ethyl recovery (**D**) % body-length of *C. elegans* in each group. Images of worms in the absence (**E**) and presence (**F**) of a 24-hour paraoxon-ethyl exposure (control and paraoxon-ethyl 500 μM) or **G** following subsequent recovery for 24-hours following paraoxon-ethyl 500 μM exposure (paraoxon-ethyl (500 μM) + Control DMSO (0.1%)). Scale bar is the same for each image.

One explanation for the recovery of *C. elegans* pharyngeal pumping following OP exposure *in vivo* is that *C. elegans* AChE is not irreversibly inhibited by OPs. However, the fact that this work has shown that a lack of recovery of AChE *in vitro* activity following exposure suggests this is unlikely. Fig. 5 showed that recovery of pharyngeal pumping is largely achieved by 6-hours, whereas over the same time frame no recovery of AChE activity is seen *in vitro*. An alternative explanation is that AChE activity *in vivo* is up-regulated, which cannot be directly measured *in vivo*. To test this possibility, a synchronised population of *C. elegans* were exposed to 500 μM paraoxon-ethyl that maximally inhibited pharyngeal pumping (Fig. 6). These worms were homogenised at the beginning of the experiment, after a 24 h exposure and following a 24-hour recovery. At each given time point, the Ellman’s assay was used to measure AChE activity at the point of maximal inhibition and after 24-hours in which recovering worms begin to recover to control-like levels of pharyngeal pumping (Izquierdo et al., 2021).

The enzyme activity extracted from intact worms exposed to paraoxon-ethyl showed that there was approximately a 50% recovery of enzyme activity *in vivo* (Fig. 5 A, 5B). This implies an intrinsic ability of intact *C. elegans* to rapidly recover enzyme activity after removal of the intact worms from AChE inhibitor, but that the enzyme *per se* is irreversibly inactivated *in vitro* (Fig. 2). In parallel, the recovery of pharyngeal pumping and body length was used as a control to confirm the previously observed recovery, which was reproduced in this assay (Fig. 6). Additionally, worm morphology partially recovers following post-exposure recovery, with worms exhibiting a more sinusoidal appearance compared to the clearly stunted morphology seen during exposure (Fig. 6E,F,G). This indicates that the observed recovery of behaviour is consistent with recovery of AChE activity in the intact worm.

N = 3–4 independent experiments, conducted in duplicate. *p < 0.05; **p < 0.01; ***p < 0.001; ****p < 0.0001, one-way ANOVA test with Bonferroni corrections.

### 3.6 Mutants with altered functionality of body wall muscle nicotinic acetylcholine receptors exhibit reduced sensitivity to nerve agent toxicity

**Fig. 7:**
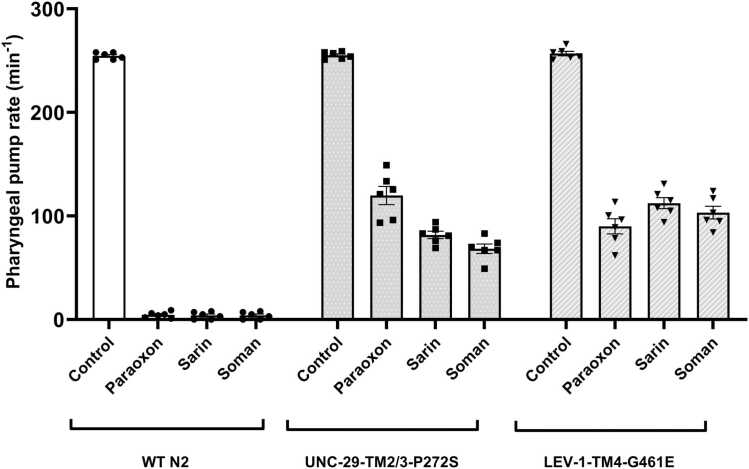
**Missense mutants of nicotinic acetylcholine receptor subunits exhibit reduced sensitivity to the nerve agents sarin and soman and the OP paraoxon-ethyl.** The missense mutants CB193 *unc-29* (*e193*), CB211 *lev-1* (*e211*) were exposed to 500 µM paraoxon-ethyl, 10 µM sarin and 50 µM soman, which maximally inhibit pharyngeal pumping in N2 worms and pharyngeal pumping was quantified at 24 h exposure. N = 3–4 independent experiments, conducted in duplicate.

It is clear that nerve agents are potent inhibitors of *C. elegans* pharyngeal pumping and that this inhibition is likely to relate to inhibition of AChE. It has previously been shown that the inhibition of pharyngeal pumping by the carbamate AChE inhibitor aldicarb is largely mediated by perturbation of cholinergic transmission at the body wall muscle (Izquierdo et al., 2021). In particular, the key determinants of this inhibition were key to cholinergic neurotransmission via the body wall muscle nicotinic acetylcholine receptor (nAChR). This was extended to paraoxon-ethyl, against which mutations altering function of the L-type nAChR conferred resistance (Izquierdo et al., 2023). As such, a limited selection of missense mutants with altered L-type nAChR functionality were screened for sensitivity to the nerve agents sarin and soman and the comparator paraoxon-ethyl. N2 and missense mutants were exposed to paraoxon-ethyl (500 μM), sarin (10 μM) and soman (50 μM), concentrations that maximally inhibit pumping in the *C. elegans*, and pharyngeal pumping was quantified at 24 h exposure (Fig. 7). Missense mutants in the body wall muscle nAChR subunits UNC-29 and LEV-1 exhibited elevated pharyngeal pumping relative to N2 at 24 h exposure in the presence of paraoxon-ethyl, sarin and soman. This suggests that nerve agents are likely to inhibit pharyngeal pumping via similar mechanisms to other OPs such as paraoxon-ethyl.

## 4 Discussion

### 4.1 *C. elegans* as a model for nerve agent intoxication

In this work, nerve agent inhibition and recovery in *C. elegans* biochemical and behavioural assays was investigated. *C.elegans* AChE was inhibited by nerve agents, albeit with a different rank order of potency to their inhibition of rat brain AChE. The lack of recovery of AChE *in vitro* is comparable with rat brain AChE and shows preservation of biochemical mechanisms between *C. elegans* and mammals. It is known that nerve agents have a greater potency relative to other OP classes to inhibit enzyme activity in guinea pig and rat brain homogenates (Pereira et al., 2014, Kovarik et al., 2025, Shih et al., 2005). This work also showed that sarin and soman were more potent in *C. elegans in vitro* and behavioural experiments compared to paraoxon-ethyl. This builds on previous investigations that compared the potencies of different OPs, but not nerve agents, in *C. elegans* and rat lysates and showed that IC_50_ values were of a comparable rank order of potency (Cole et al., 2004). It is important to note that there are some differences, where at the enzyme level *C. elegans* AChE was less sensitive to all of the nerve agents, relative to rat brain AChE. Despite this, there was a complete inhibition of AChE activity in the presence of all of the agents at increasing concentrations.

In *C. elegans*, there are 3 forms of AChE which are encoded by three genes: *ace-1, ace-2* and *ace-3* (Combes et al., 2001, Selkirk et al., 2005). This is different to the mammalian brain, which contains monomeric and tetrameric forms of AChE protein derived from the same gene. Further, mammals also express butyrylcholinesterase, which is not found in *C. elegans*. In the context of homogenate measurement, in *C. elegans*, 95% of the activity measured is dependent on *ace- 1* and *ace- 2* and the remaining 5% on *ace-3* (Combes et al., 2001). In particular, the activity of the different isoforms of *C. elegans* vary. In some ways, the *ace-3* isoform in *C. elegans* acts similarly to butrylcholinesterase (BuChE) in the mammalian brain. This is because *ace-3* accounts for a small contribution to AChE activity, like that in mammals where butyrylcholinesterase accounts for 10% of the activity in the brain. The three different genes in *C. elegans* are different to rat brain AChE, which has different forms that are made up by a singular AChE gene. Although the inhibition of AChE in *C. elegans* by some OPs may be different from that in mammalian species, the *C. elegans* isoforms of AChE are still subject to a potent and complete inhibition. The complete inhibition of *in vitro* activity in *C. elegans* suggests that the measurement of homogenate AChE will be dominated by activity from *ace-1* and *ace-2* and is therefore a useful biochemical proxy for mammalian biochemical observations.

### 4.2 *C. elegans* exhibited a reduced sensitivity to VX *in vivo* and *in vitro* relative to mammals

In this work, an unexpected observation was a reduced sensitivity of the *C. elegans* AChE to VX. This is unlike mammalian species, where VX has a greater potency to inhibit AChE *in vitro* relative to other OPs tested here (Worek et al., 2020). The array of chemical characteristics displayed by OPs results in differences that account for changes in bioavailability, absorption, distribution, degree of AChE inhibition, metabolism and elimination (Hutson and Millburn, 1991, Wallace, 1992). The difference in the VX sensitivity could be a result of 1) pharmacodynamic parameters such as AChE structural differences between species affecting VX binding, 2) pharmacokinetic differences as a result of the physicochemical properties of VX relative to sarin and soman, or 3) the means of exposure, as the internal dose the worm receives was not quantified, nor is it known whether the inhibitors are stable in the aqueous media or NGM. The altered VX sensitivity could also be a combination of the above. The combination of a reduced sensitivity to VX seen in *in vitro* AChE activity assays, not just *in vivo* pharyngeal pumping, does however suggest that the reduced sensitivity is mostly at the level of enzyme activity.

A previous report suggested that *C. elegans* is less sensitive to OPs containing a thiol-group such as demeton-*S*-methylsulfone (Cole et al., 2004). This suggest that structural differences between sarin and VX affect the interaction with *C. elegans* AChE and may be a key determinant underlying the reduced susceptibility of *C. elegans* AChE and *in vivo* to VX relative to other OPs. The compounds in this previous study exhibited a reduced relative toxicity in *in vitro* and behavioural experiments and the authors suggested this as a result of their thioester containing bond in their structure, or the size of the molecules that stop thiol containing OP from binding to AChE. It is currently unclear why *C. elegans* are less susceptible to certain OP structures at a biochemical and behavioural level, however it is interesting that VX also exhibits a similar phenomenon in this work as previously reported (Cole et al., 2004).

Considering *C. elegans* AChE is less sensitive to inhibition by VX than rat brain AChE, it could be postulated that the structure of *C. elegans* AChE may result in a reduced sensitivity to VX. A simple alignment of the AChE sequences of mammals (rat and human) with that of *C. elegans* showed they have relatively low sequence identity. Despite this, there is good conservation in the critical elements that underpin AChE activity such as the catalytic triad, acyl pocket or oxyanion hole (Supplementary Figure 1) (Selkirk et al., 2005). This suggests that the differences amongst the species likely reside outside the fundamental regions that allow enzyme function. In *Drosophila melanogaster,* it has been observed that the residues that make up the gorge also exhibit a low sequence identity to mammalian species and the AChE possessed a narrower gorge (Harel et al., 2000). This suggests that *C. elegans,* like *D. melanogaster*, may exhibit a different structure outside of the fundamental regions that could impair access to the enzyme’s binding site by larger molecules such as VX. Taken together, the combination of the structural differences between *C. elegans* and mammalian acetylcholinesterase and the thiol-group containing VX, might provide an explanation for the reduced sensitivity of *C. elegans* in *in vitro* and *in vivo* experiments to VX in this study.

### 4.3 Pharyngeal pumping as a measure of recovery of biochemical AChE activity

Pharyngeal pumping spontaneously recovered following removal from OP exposure, despite the observed lack of spontaneous recovery of AChE activity *in vitro* even after 6-hours*.* This is surprising, as OPs irreversibly inhibited AChE activity *in vitro*. Paraoxon-ethyl and sarin exhibited a faster level of recovery, about 90% after 2-hours, compared to soman and VX which exhibited a slower recovery of 80% at 24-hours.

It was hypothesised that paraoxon-ethyl and sarin may have a faster recovery because of reduced re-inhibition of newly synthesised enzyme relative to VX and soman. This hypothesis was further investigated by comparing the whole organism phenotype in exposed and recovering worms, before going on to measure the extracted enzyme activity from worms following full inhibition and increasing times into recovery. The clear differences in *in vitro* and *in vivo* recovery suggest that additional parameters within the whole organism differ to the *in vitro* experiment. This could result from a faster enzyme turnover rate in the worm compared to mammals, which allows *C. elegans* to be able to recover affected behaviours. For example, it is known that the AChE enzyme in chick cells has a biogenesis of a few hours (Rotundo, 1988, Rotundo, 2017), a further 3-hours is needed from synthesis to secretion to the plasma membrane (Rotundo, 1984). Despite this, around 80% of the newly synthesised enzyme is catalytically inactive AChE, which has been observed in chicken nerves, muscle and human brain (Campanari et al., 2014, Chatel et al., 1993). In addition, the remaining 20% of the newly synthesised enzyme exists as an unstable form of AChE (prone to degradation by proteases and heat) that is catalytically active but requires further maturation with non-catalytic subunits to allow AChE assembly (Eichler et al., 1995). The turnover rate of *C. elegans* AChE is currently unknown, but the ability of the worm to recover from OPs after 2-hours suggests that the enzyme turnover rate may be faster than in mammals.

The fact that the *C. elegans* behaviour recovers to 90% following maximal OP exposure suggests that maximal enzyme activity is not needed to recover function of the neuromuscular junction. This process is part of the safety factor of the ability of the neuromuscular junction to remain effective under various physiological conditions and stresses by having an excess of AChE (Wood, Slater, 2001). Despite this, the lack of stable and active enzyme is associated with a slow clinical recovery in mammals following OP exposure. In this work, despite there only being a 50% recovery of enzyme activity, *C. elegans* was able to recover behaviour to a greater extent. This is exemplified in body-length and pharyngeal pumping which recovers to around 80%. The recovery of pharyngeal pumping after exposure could suggest that there is early enzyme biogenesis. In fact, it may suggest that unstable and immature, yet catalytically active enzyme, is able to recover enough activity in *C. elegans* to allow functional or physiological recovery.

### 4.4 The effects of nerve agents on pharyngeal pumping are likely mediated by distal effects on body wall muscle cholinergic signalling

Following on from previous work, where it was shown that the effects of aldicarb and paraoxon-ethyl on pharyngeal pumping were mediated by distal inhibition via body wall muscle nAChRs, this was extended to the nerve agents sarin and soman. The mutants CB193 *unc-29* (*e193*), CB211 *lev-1* (*e211*) exhibited a reduced inhibition of pharyngeal pumping at 24 h exposure relative to N2 in the presence of sarin, soman and paraoxon-ethyl. This is indicative of a shared mechanism of inhibition of pharyngeal pumping by nerve agents and other OPs, likely via excessive signalling at the body wall muscle nAChR. This does however require deeper investigation, through further mutant screening and pharmacological manipulation. The previous work showed these mutants UNC-29-TM2/3-P272S and LEV-1-TM4-G461E reduced receptor function in electrophysiological recordings (Izquierdo et al., 2023). This indicates that the reduced sensitivity exhibited by these missense mutants is likely a result of reduced receptor function. This suggests that disruption of the *C. elegans* body wall muscle nAChR during OP exposure by altering its sensitivity could hold relevance to the development of new treatments to human OP and nerve agent exposure, though further investigation is required (Izquierdo et al., 2022, 2023).

### 4.5 Limitations

There are limitations to the described study, which need to be acknowledged. It is recognised that whilst the described work serves to benchmark *C. elegans* as a model organism for research into the toxic effects of nerve agents, there remains a need to more deeply explore the mechanisms underlying nerve agent effects on *C. elegans* behaviours. Whilst the experiments with missense mutants suggest a potential mechanism for nerve agent-induced inhibition of pharyngeal pumping, these were limited and should be investigated further. This also extends to the observed reduced sensitivity of *C. elegans* at both the *in vivo* and the enzymatic level to VX relative to the other nerve agents tested here. It is recognised that the reduced sensitivity of *C. elegans* to VX could limit some aspects of the translational potential of this model This reinforces a need to adopt of multi-model approach to the investigation of the toxicity of compounds such as nerve agents. The altered sensitivity of *C. elegans* to VX should be explored further, which could include expanding the range of compounds tested to other OPs, including other V-series nerve agents (e.g. VM). Furthermore, it is recognised that the observations in this paper focus on behavioural bioassays and measurements of enzymatic function. This does not preclude other effects of the tested compounds, for example cytotoxicity, and these should be investigated further, though the behavioural recovery seen suggests limited off-target effects,

## 5 Conclusion

Overall, this work has shown that *C. elegans* has the potential to serve as a non-mammalian biochemical and *in vivo* model for nerve agent exposure studies. It has clear potential for investigating toxicology and investigating new treatments for OP (Dabholkar et al., 2023, Eddleston et al., 2002) and nerve agent poisoning (Voros et al., 2024). This is supported by the observed similar rank order of potency between the nerve agents sarin and soman relative to paraoxon-ethyl that can be measured at the enzyme level. This rank order of potency was the same in the *C. elegans* behaviour pharyngeal pumping, which supports this behaviour as a proxy for enzyme inhibition. This study also demonstrates that paraoxon-ethyl has sufficient key similarities in inhibition and recovery to nerve agents and is a valid surrogate for nerve agent exposure. The identified deviations of the model, such as a reduced VX sensitivity, allows for a comparative approach to inform on the determinants of nerve agent model of action. The altered sensitivity to VX does however suggest differential sensitivity of *C. elegans* to G-series and V-series agents that requires further investigation to better understand the strengths and limitations of *C. elegans* as a model for nerve agent exposure research. The important observations in this work are 1) *in vitro* biochemical AChE activity can be a read out of *in vivo* behavioural observations using pharyngeal pumping to better understand fundamental mechanisms of nerve agent exposure and recovery and 2) the model and observations suggest that relatively rapid recovery of function is most likely due to AChE synthesis and turnover. The second observation may open new routes to mitigation based on this recovery mechanism, especially with *C. elegans* having a capability to retain recoverable biological function under conditions where survival would not be possible in other models. Altogether, this study further supports the use of *C. elegans* as a flexible model for nerve agent investigations.

## CRediT authorship contribution statement

**James Kearn:** Writing – review & editing, Writing – original draft, Visualization, Supervision, Project administration, Methodology, Investigation, Funding acquisition, Formal analysis, Data curation. **Vincent O′Connor:** Writing – review & editing, Writing – original draft, Visualization, Supervision, Project administration, Methodology, Investigation, Funding acquisition, Conceptualization. **Lindy Holden-Dye:** Writing – review & editing, Writing – original draft, Visualization, Supervision, Project administration, Methodology, Investigation, Funding acquisition, Conceptualization. **A. Christopher Green:** Writing – review & editing, Writing – original draft, Visualization, Supervision, Project administration, Investigation, Funding acquisition, Conceptualization. **Johanna Haszczyn:** Writing – original draft, Visualization, Validation, Methodology, Investigation, Formal analysis, Data curation, Conceptualization.

## Funding

This study was jointly funded by the UK Ministry of Defence and the South Coast Biosciences Doctoral Training Partnership, via the Biotechnology and Biological Sciences Research Council (BBSRC). The design, performance, data interpretation and manuscript writing were solely under the control of the authors.

## Declaration of Competing Interest

The authors declare that they have no known competing financial interests or personal relationships that could have appeared to influence the work reported in this paper.

## Data Availability

Data will be made available on request.
