# Supplementary material for: An investigation into the use of Caenorhabditis elegans as a model organism for nerve agent exposure research
Source: Neurotoxicology. 2026 May;114:None. doi: 10.1016/j.neuro.2026.103453 (PMC13268407; doi:10.1016/j.neuro.2026.103453)
Supplement: Supplementary file 1 — Supplementary material [file mmc1.docx]

**Supplementary Figures**


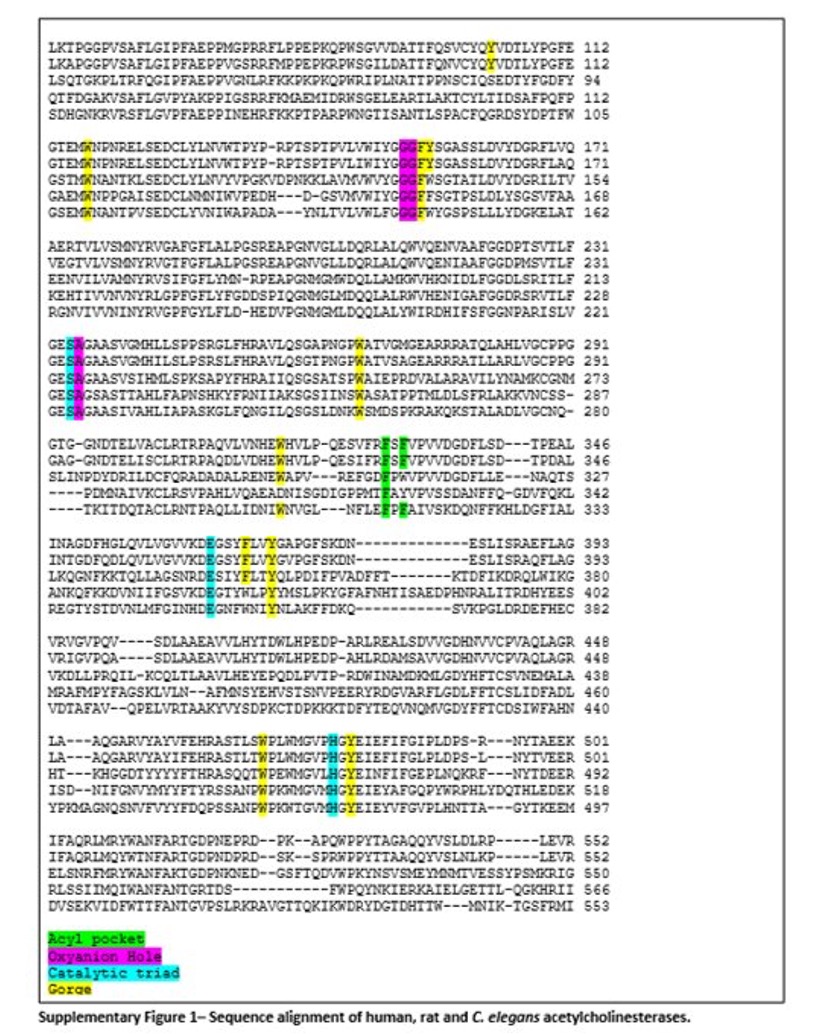
**Supplementary Figure 1– Structural differences between mammalian and *C. elegans* AChE sequences and structures.** Uniprot protein codes for *Homo sapiens* (P22303), *Rattus rattus* (P37136), *C. elegans (ace-1)* (P38433), *C. elegans (ace-2)* (G5EDV9) and *C. elegans (ace-3)* (Q9U295) (in order from top to bottom). Fundamental regions of the enzyme are colour coded. Gorge (yellow), acyl pocket (green), catalytic triad (blue) and oxyanion hole (pink). Residues were selected based on the evidence from (Dvir et al., 2010; Silman & Sussman, 2008).
